# Up-Regulation of Human Inducible Nitric Oxide Synthase by p300 Transcriptional Complex

**DOI:** 10.1371/journal.pone.0146640

**Published:** 2016-01-11

**Authors:** Zhong Guo, Liang Zheng, Xinghua Liao, David Geller

**Affiliations:** 1 Department of Medicine, University of Pittsburgh, Pittsburgh, Pennsylvania, 15261, United States of America; 2 Department of Surgery, University of Pittsburgh, Pittsburgh, Pennsylvania, 15261, United States of America; Universidade de São Paulo, BRAZIL

## Abstract

p300, a ubiquitous transcription coactivator, plays an important role in gene activation. Our previous work demonstrated that human inducible nitric oxide synthase (hiNOS) expression can be highly induced with the cytokine mixture (CM) of TNF-α + IL-1β + IFN-γ. In this study, we investigated the functional role of p300 in the regulation of hiNOS gene expression. Our initial data showed that overexpression of p300 significantly increased the basal and cytokine-induced hiNOS promoter activities in A549 cells. Interestingly, p300 activated cytokine-induced hiNOS transcriptional activity was completely abrogated by deleting the upstream hiNOS enhancer at -5 kb to -6 kb in the promoter. Furthermore, p300 over-expression increased cytokine-induced transcriptional activity on a heterologous minimal TK promoter with the same hiNOS enhancer. Site-directed mutagenesis of the hiNOS AP-1 motifs revealed that an intact upstream (-5.3kb) AP-1 binding site was critical for p300 mediated cytokine-induced hiNOS transcription. Furthermore, our ChIP analysis demonstrated that p300 was binding to Jun D and Fra-2 proteins at -5.3 kb AP-1 binding site *in vivo*. Lastly, our 3C assay was able to detect a long DNA loop between the hiNOS enhancer and core promoter site, and ChIP loop assay confirmed that p300 binds to AP-1 and RNA pol II proteins. Overall, our results suggest that coactivator p300 mediates cytokine-induced hiNOS transactivation by forming a distant DNA loop between its enhancer and core promoter region.

## Introduction

The expression of inducible nitric oxide synthase (iNOS) can be activated by immunologic and inflammatory stimuli such as cytokines or lipopolysaccharide (LPS) in different types of cells and tissues [1], [2]. The expression of human inducible nitric oxide synthase (hiNOS) gene in primary human hepatocytes was originally identified by stimulating with a cytokine mixture (CM) of TNF-α, IL-1β, IFN-γ, and LPS [3]. Subsequently, the human iNOS gene was cloned from LPS and CM-stimulated primary human hepatocytes [4]. The molecular regulation of the human iNOS gene is complex and is mostly regulated at transcriptional level. The functional promoter region of hiNOS gene can extend to16 kb in length [5]. There are numerous transcription factors such as AP-1, C/EBP, CREB, GATA, HIF, IRF-1, KLF6, NF-AT, NF-κB, NRF, Oct-1, PARP1, p53, Sp1, STAT-1α, TBE, and TCF, which were validated to bind hiNOS promoter [6], [7], [8], [9], [10], [11], [12].[13]. There is also a classical cytokine-induced enhancer between -5 and -6 kb of human iNOS promoter [14]. The human iNOS core promoter contains a TATA box about 24 bp from the transcription start site. Near the TATA box, it also contains multiple binding sites for the transcription factors NF-κB, and C/EBP β [13].

In previous studies, p300, a transcriptional coactivator had been shown to be important for transactivation of the murine iNOS promoter. LPS/IFN-γ induced p300 binding and iNOS promoter activity. p300 interacted with downstream NF-κB and AP-1 motifs in the -1.5 kb murine iNOS promoter [15]. However, the human iNOS promoter has been shown to span -10 kb and is far more complex than the rodent iNOS promoter. Functionally important NF-κB-like sequences have been identified at –5.5, –5.8, –6.1 kb, and –8.2 kb in the human iNOS promoter. There are also two functional AP -1 binding sites at –5.1 and –5.3 kb [16], [17]. Active IFN-induced STAT1 binding sites are seen at -5.2 and -5.8 kb in the hiNOS promoter [8]. A far-upstream functional Oct-1 motif was also identified at -10.2 kb in the human iNOS promoter that regulates cytokine-induced human iNOS gene transcription [12]. While specific cytokine-induced trans-acting transcription factors binding to cis-acting DNA motifs have been identified, there is no information regarding a role for p300 or other captivators in regulating hiNOS gene transcription. In this study, we investigated whether p300 mediates trans-activation of the human iNOS gene and further examine which transcription factors are involved.

## Materials and Methods

The University of Pittsburgh institutional review board (IRB) approved this study. The IRB approval number is PRO1210076. The approval protocol title is “Liver Tissue and Cell Distribution System (LTCDS)”. Human hepatocytes were isolated from histological normal operative wedge resections by using collagenase perfusion. In detail, hepatocytes were separated from non-parenchymal cells by differential centrifugation four times at 50xg. The hepatocytes were then further purified over a 30% Percoll gradient at a concentration of 1 million hepatocytes per ml of Percoll to obtain a highly purified cell population. Hepatocyte purity by microscopy was >98% and viability consistently exceeded 95% by trypan blue exclusion. We confirm that any donors of hepatocytes used for this study provided written informed consent.

### Materials

Human recombinant TNF-α, IL-1β and IFN-γ proteins were purchased from R&D Systems. Lipofectamine 2000 was obtained from Life technologies. Antibodies against p300, AP-1 and polymerase II were acquired from Santa Cruz Biotechnology. All other reagents were obtained from Sigma.

### Plasmids

The p300 expression vector pcDNA3.1-p300 was purchased from Addgene. The human iNOS promoter reporter plasmid vector piNOS (7.2)luc contains -7.2 kb of upstream 5’-flanking DNA linked to the luciferase reporter gene and have been described previously [5], [18]. The mutated constructs corresponding to AP-1 binding sites at -5.3 or -5.1 kb of hiNOS promoter were generated by using the QuickChange mutagenesis kit from Stratagene. The mutant DNA oligonucleotides were as follows: mutated sequences are underlined. Pr8-1u, 5′-CCAGCTTCCGTAACACTC-3′; Pr8-1d, 5′-TTTGTGTCCGTAACGCCC-3′. Confirmation of the mutant constructs was accomplished with DNA sequencing analysis by the University of Pittsburgh Sequencing Facility.

### Cell Culture

The A549 cell line was obtained from the ATCC and cultured in medium as previously described [9]. Primary human hepatocytes were obtained from freshly resected human liver specimens under IRB approved protocol. 3 × 10^6^ human hepatocytes were grown in DMEM with 10% low endotoxin FBS, 100 units/ml penicillin, 100 μg/ml streptomycin, 2 mM l-glutamine and 15 mM Hepes, pH 7.4. Cells were plated onto 6 well cell culture plates (Corning) and stimulated with a cytokine mixture (CM) of TNF-α (1,000 units/ml) + IL-1 β(100 units/ml) + IFN-γ(250 units/ml).

### Transfections and Reporter Gene Assays

DNA plasmid constructs were transfected into cells in six-well plates (Corning,), using Lipofectamine 2000 for human cells. Cells were exposed to serum-free medium containing 1 μg DNA of different hiNOS promoter constructs with 10 μg of liposomes for 6 h, washed, and replaced with medium supplemented with 5% calf serum. Cells were lysed with Dual-Glo luciferase Assay System (Promega). Luciferase activity was examined by AutoLumat LB 953 luminometer (Berthold).

Control siRNA (sc-37007) and siRNA against p300 (sc-29431) were obtained from Santa Cruz Biotechnology, and cells were transfected according to the manufacturer's instruction. About 2 × 10^6^ cells were seeded on 6 well cell culture plates one prior to the day of transfection and grew to 60–80% confluent. For each transfection, 50 pmols control siRNA or p300 siRNA were added in siRNA transfection medium. Cells were treated with siRNA medium for 5 hours. The medium were aspirated and replaced with normal growth medium. The sequence of p300 siRNA (sc-29431) is: CCCCUCCUCUUCAGCACCA. The p300 siRNA (sc-29431) was validated in A549 cells by Western Blot (S1 Fig).

### Western Blot and PCR

Protein extraction and western blot analysis and PCR assay were performed as previously described [8]. The following primers were used for human iNOS mRNA expression: 5′-CAGCGGGATGACTTTCCAA-3′ (forward) and 5′-AGGCAAGATTTGGACCTGCA-3′ (reverse); human β-actin iNOS primers, 5′-AGGCATCCTCACCCTGAAGTA-3′ (forward) and 5′-CACACGCAGCTCATTGTAGA-3′ (reverse). Amplified DNA fragments were analyzed on a 1% agarose gel by electrophoresis.

### NO production assessment

Culture supernatants were collected and assayed for nitrite, a stable end product of NO oxidation, using the Griess reaction as described [19].

### Chromatin immunoprecipitation (ChIP) assay and qPCR

Hepatocytes was treated with CM for 2 h. The ChIP assay is performed following the recommendations of Upstate Biotechnology. Formaldehyde was added the culture medium at a final concentration of 1% to freeze the DNA-protein and protein-protein interactions. Cells were washed twice with ice-cold PBS, and further resuspended in cell lysis buffer (5 mM Pipes, pH 8.0; 85 mM KCl; and 0.5% Nonidet P-40) containing 0.5 mM PMSF and kept on ice for 15 min. Then cell lysates were sonicated on ice until the cross-linked chromatins were sheared to yield DNA fragments between 200 bp and 1 kb. The supernatants were immunoprecipitated with normal IgG, anti-Jun B Ab, anti-c-Jun Ab, anti-Jun D Ab, anti-Fra 2 Ab, anti-p300 Ab or anti-p65 with protein G-agarose beads at 4°C overnight. These supernatants were added 5M NaCl and heated at 65°C to reverse histone-DNA crosslinks. The immunocomplexes were further treated with DNase- and RNase-free proteinase K, and DNA is purified using a DNA purification kit (Qiagen). Quantitative PCR was carried on the StepOne Plus real-time PCR system (Applied Biosystems) by using the threshold cycle method of calculating relative ChIP DNA products expression. The following primers were used for ChIP assay: AP-1u primers, 5′-TTCTGGGGAGGCTTGACAAG -3′ and 5′-GAAGTGAAGTGAAGGGATTT-3′; AP-1d primers, 5′-AAATCCCTTCACTTCACTTC-3′ and 5′-CCGTGAGCCCTATGTCATTT-3′.

### Chromosome Conformation Capture (3C) Assay

Human hepatocytes (1 × 10^7^) were grown in 10-cm dishes and added with 1% formaldehyde for 10 min at room temperature. About 2.5 ml of 2.5 M glycine were added to quench the formaldehyde and stop the crosslinking. The cells were resuspend in 1 ml ice-cold lysis buffer, consisting of 10 mM Tris,pH 8, 10 mM NaCl, 0.2% Igepal (NP-40) and protease inhibitors (2 μg/ml leupeptin, 2 μg/ml aprotinin and 2 μg/ml pepstatin). Lyse cells were sonicated on ice, and spun down (5 min, 5000 rpm). DNA fragments from the cross-linked chromatins were further digested by the restriction enzyme Hind III overnight at 37°C. T4 DNA ligase enzymes were added into the reaction with ligase buffer with 0.1% SDS and 1% Triton X-100, and further incubated at 16°C overnight. The cross-links were reversed by incubation of 10 μg/ml proteinase K at 65°C for 5 hours. The DNA was purified by phenol-chloroform extraction and ethanol precipitation. These DNAs were quantified and used as a PCR temple. Amplified DNA fragments are analyzed on a 1% agarose gel by electrophoresis. The sequences of primers are listed as followed: primer A: 5′- GCTTGACAAGAAACGAGGCT-3′; primer B primer: 5′- GGCCTCTGAGATGTTGGTCT-3′. About 145 bp PCR products were confirmed by sequencing as predicted for the truncated human iNOS DNA fragment with Hind III site.

### ChIP Loop Assay

Formaldehyde is added the culture medium at a final concentration of 1% to freeze the DNA-protein and protein-protein interactions. Cells were washed twice with ice-cold PBS, resuspended in cell lysis buffer (10 mM Tris,pH 8, 10 mM NaCl, 0.2% Igepal) and kept on ice for 15 min. Then cell lysates are sonicated on ice and digested by the restriction enzyme Hind III. The chromatin fragments were immunoprecipitated with normal rabbit IgG, anti-AP-1 Ab, anti-RNA pol II Ab, or anti-p300 Ab with protein G-agarose beads at 4°C overnight. T4 DNA ligase enzymes were added into the reaction with ligase buffer with 0.1% SDS and 1% Triton X-100, and further incubated at 16°C overnight. The cross-links were reversed by incubation of 10 μg/ml proteinase K at 65°C for 5 hours. DNA was purified using a DNA purification kit (Qiagen). PCR assay was performed as described above.

### Statistical analysis

Results were given as means ± SD. Comparisons versus controls were performed using ANOVA followed by the Tukey’s multiple comparison method as our post-hoc test. P values less than 0.05 were considered significant.

## Results

### No obvious increase on endogenous p300 protein expression by cytokine stimulation

**Fig 1:**
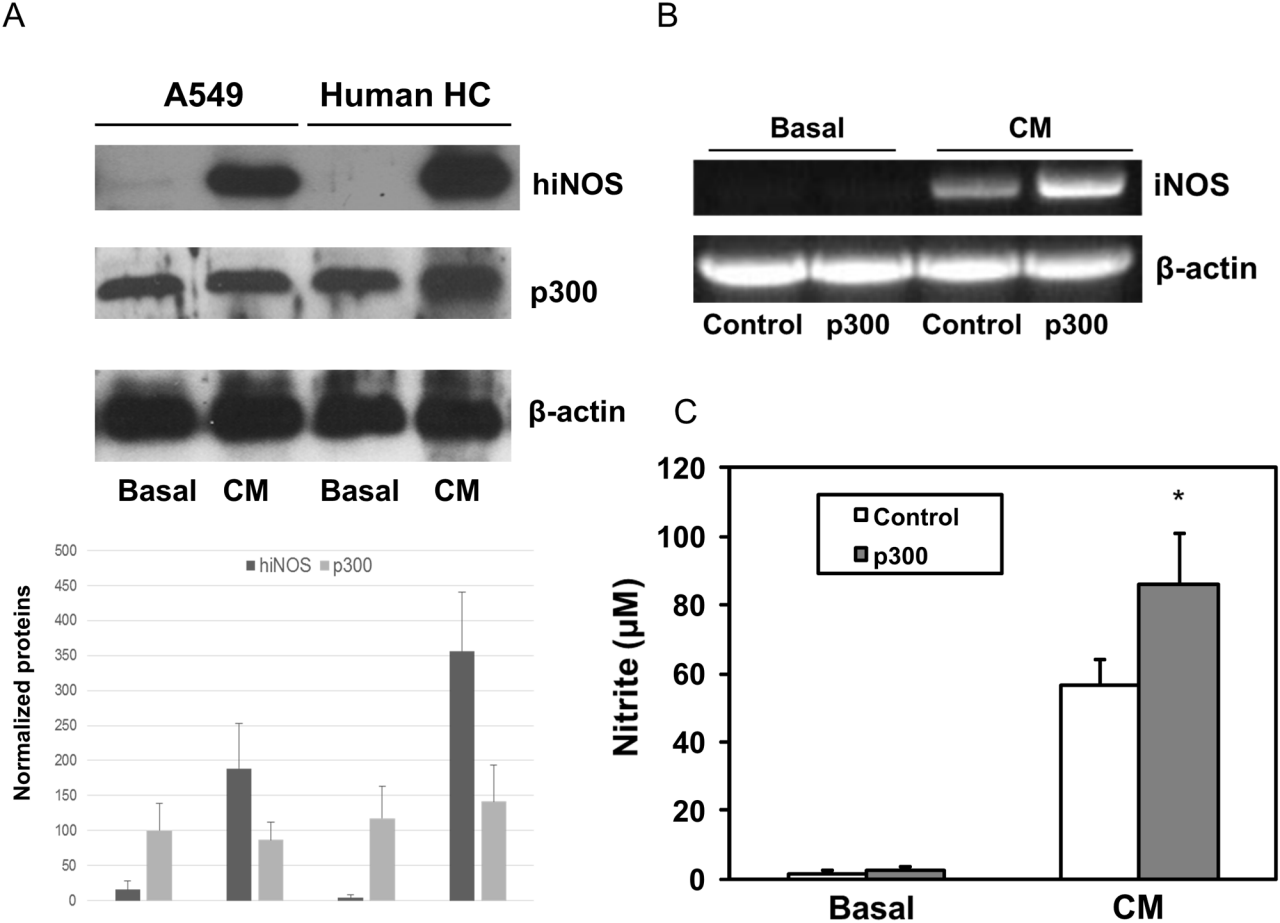
Effect of p300 on cytokine induced hiNOS expression. (A) Western blot of cytokine mix (CM) TNF-α + IL-1β + IFN-γ induced hiNOS protein, but not p300 nuclear proteins in human hepatocytes and A549 cells. Three similar Western blot experiments were quantified for hiNOS and p300 proteins. (B) RT-PCR analysis of hiNOS mRNA expression in human hepatocytes after overexpression of p300. Hepatocytes were transfected with p300 expression vector or control empty vector, and then treated with CM. mRNAs were extracted from hepatocytes after CM treatment for 6 hr. (C) Griess assay of NO produce in human hepatocytes. Medium from cell culture was collected from hepatocytes after CM treatment for 24 hr. The graph shows means ± SD.

Previously, we observed that hiNOS gene was strongly induced with the cytokine mixture (CM) of TNF-α, IL-1β and IFN-γ. However, whether p300 can also be induced by cytokines is unknown. Therefore, we performed Western blot experiments to assess p300 nuclear protein expression in response to CM in human A549 lung epithelial cells cell and primary human hepatocytes (HC). As depicted in Fig 1A, human iNOS protein can be strongly induced by CM in both A549 and primary human HC. Basal hiNOS protein was very weak or hardly detected in resting cells. In contrast, p300 protein was expressed constitutively in both the A549 cells and the primary human HC. With CM stimulation, there is no significant change in level of p300 nuclear protein in A549 cells, and minimal change in primary human HC. These data suggest that cytokines can activate endogenous hiNOS expression, but not p300 nuclear proteins.

### Up-regulation of human iNOS transcription by p300

To investigate whether p300 can trans-activate the human iNOS gene, we transiently transfected human HC with p300 expression vector. Empty plasmid vector served as negative control. Following DNA plasmid transfection, primary human HC were treated with CM. After 6 hour treatment, total RNA were collected for mRNA analysis. CM induced hiNOS mRNA expression by RT-PCR, while hiNOS mRNA was not observed in resting HC (Fig 1B). Overexpression of p300 further increased CM-induced hiNOS mRNA transcripts by RT-PCR, but alone did not induce the detectable hiNOS mRNA at basal level (Fig 1B). The p300 –mediated increase in stimulated hiNOS mRNA also resulted in increased NO synthesis of nitrite, detected by Griess assay of culture supernatants after 24 hour CM stimulation (Fig 1C). In order to demonstrate a physiological role for endogenous p300 in hiNOS transactivation, we performed qPCR assay to dectect hiNOS expersion after knockdown of p300 endogenous expression. Inhibition of endogenous p300 expression with p300-siRNA (but not scrambled control siRNA) decreased the CM-induced human iNOS mRNA expression by least 50%, but no significant effect on basal level was observed (S2 Fig).

### The upstream enhancer at -5 kb to -6 kb in the hiNOS promoter is necessary for p300 transactivation

**Fig 2:**
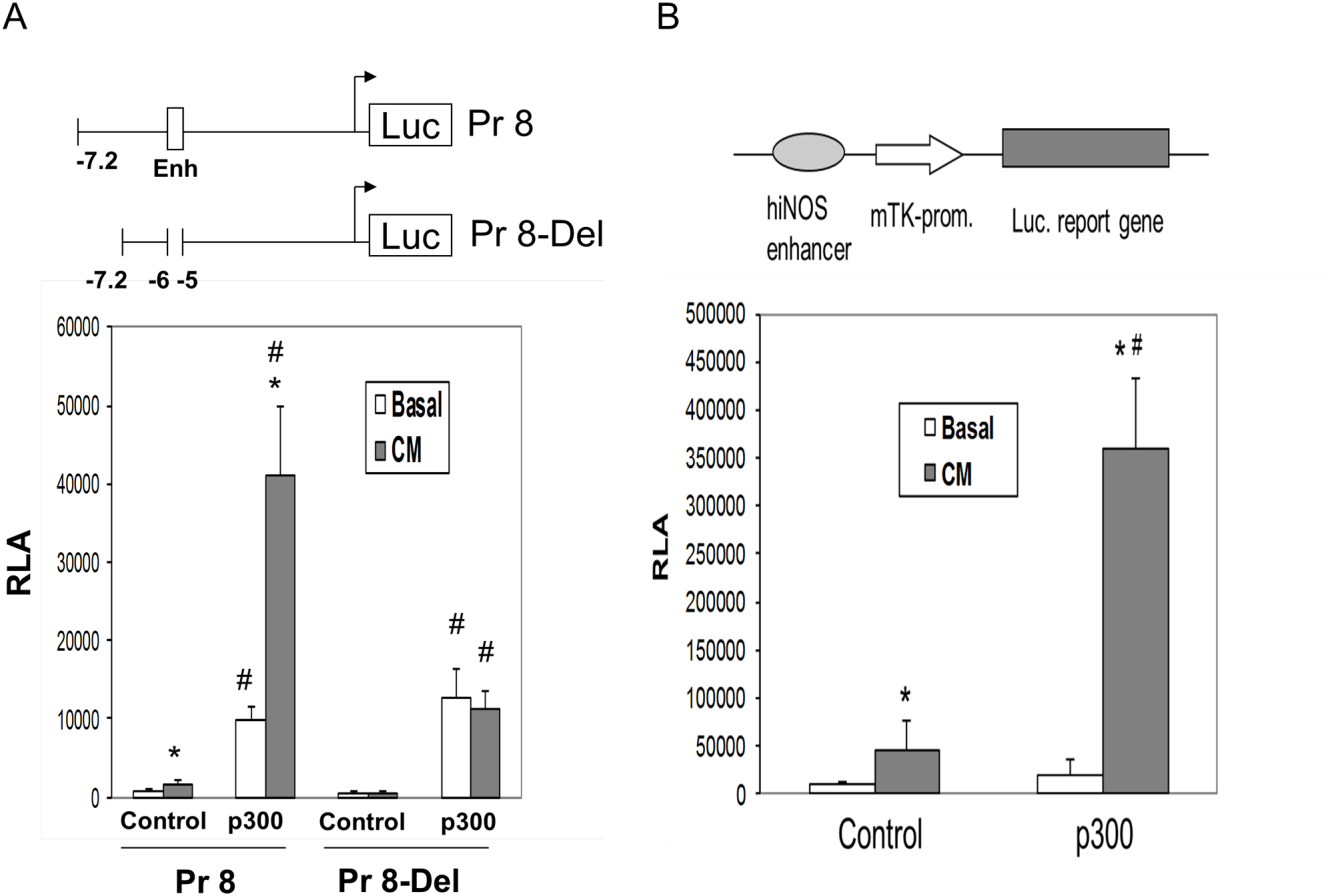
p300 mediated transactivation of the hiNOS or heterologous promoter. (A) The –7.2 kb wild-type (WT) human iNOS promoter construct (Pr8) or deleted -5 to -6 kb enhancer region (Pr8-Del), were co-transfected into A549 cells with p300 expression vector. Basal and stimulated luciferase activities were determined 6 hours after cytokine mix (CM) stimulation. Relative luciferase activities (RLA) values are the means ± sd of at least three separate experiments performed in triplicate. *Indicates *P* < 0.05 *vs*. basal, # indicates P < 0.05 vs. control (B) The minimal TK promoter construct with ligated hiNOS enhancer was co-transfected into A549 cells with p300 expression vector. Co-transfection with empty vector served as control. Basal and stimulated luciferase activity was determined 6 hr after cytokine stimulation. Values shown are the means ± sd of at least three separate experiments performed in triplicate. *Indicates P < 0.05 vs. basal, # indicates P < 0.05 vs. control.

Previously we identified that upstream -7.2 kb of the hiNOS promoter was required for significant transcriptional activation [5], and that a classical cytokine-induced enhancer resided between -5 kb and -6 kb in the hiNOS promoter [14]. To determine the effect of p300 on transactivation of the hiNOS promoter, we co-transfected the -7.2 kb hiNOS promoter construct (labeled Pr8) along with p300 expression vector, and determined luciferase reporter activity in resting and CM-stimulated human A549 cells (Fig 2A). CM alone induced the similar 4-fold increase above basal activity in Pr8 vector that we have previously shown, while co-transfection with p300 super-induced a 19-fold dramatic increase in hiNOS promoter activity compared to empty vector control (Fig 2A). Interestingly, the ratio or fold-increase in CM-induced hiNOS promoter activity remained at 4-fold with or without p300 co-transfection. Over-expression of p300 also elicited a modest increase in basal hiNOS promoter activity compared to empty vector control.

Next, to localize where in the -7.2 kb hiNOS promoter p300 was transactivating, we also examined the effect of p300 on the cytokine-induced enhancer region at -5 kb to -6 kb in the hiNOS promoter. When the enhancer region at -5 kb to -6 kb was deleted from the hiNOS promoter (Pr 8–Del), all CM-induced activity was lost, and the ability of p300 to super-induce hiNOS promoter activity was also abrogated (Fig 2A). These findings indicate that the cytokine-induced enhancer region at -5 kb to -6 kb is required for p300-mediated increase in CM-induced hiNOS promoter activity. However, the p300-mediated increase in basal hiNOS promoter activity was preserved and indicates the upstream enhancer region at -5 kb is not required for basal effects.

### p300 confers transactivation to a heterologous minimal promoter

To validate that p300 is capable of mediating transactivation to a heterologous minimal promoter with the same hiNOS enhancer region, we ligated the hiNOS enhancer in front of the minimal herpes thymidine kinase (TK) reporter and transfected the A549 cells with or without the p300 expression vector. CM alone significantly increased activity of the TK reporter that contained the hiNOS enhancer compared to basal levels, and co-transfection of p300 dramatically increased CM inducibility compared to control vector (Fig 2B). These findings show that p300 can also drive CM-induced transcriptional activation on a heterologous promoter dependent on the presence of the hiNOS enhancer.

### Functional interaction of AP-1 sites at hiNOS enhancer with p300

**Fig 3:**
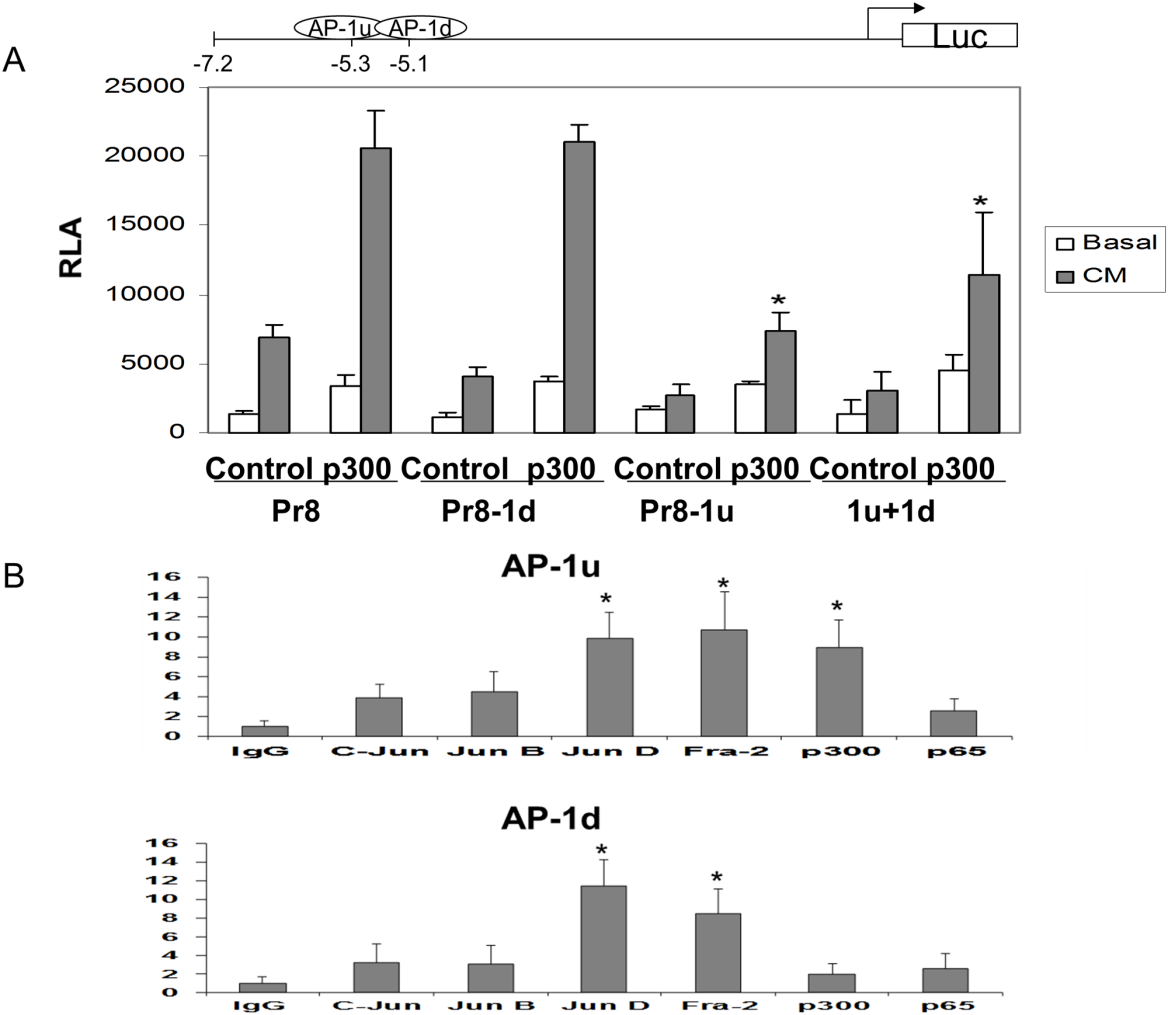
In vitro and In vivo analysis of AP-1 binding sites in the hiNOS enhancer. (A) Mutagenesis analysis of AP-1 sites at -5.1 kb downstream (Pr8-1d) or -5.3 kb upstream (Pr8-1u) in the hiNOS promoter. Mutant construct for each site or double AP-1 mutant (1u+1d) were generated in the hiNOS Pr8 promoter luciferase reporter plasmid driven by pCMV promoter. Wild-type hiNOS promoter luciferase reporter plasmid served as control. * Indicates p <0.05 vs. p300. (B) ChIP analysis of AP-1 binding sites in the hiNOS enhancer with various antibodies. * Indicates p <0.05 vs. Ig G.

We and others have identified that the upstream enhancer region at -5 kb in the hiNOS promoter contains multiple functional transcription factor binding sites including NF-κB, Stat-1, and AP-1 sites. To determine which DNA response elements were participating in p300-mediated transactivation, we generated selective site-directed mutant reported constructs changing 3 nucleotides in each response element. Then, A549 human lung cells were transiently transfected with the -7.2 kb wild-type human iNOS promoter luciferase construct (Pr8) as well as each mutant construct. Promoter activities were measured as relative luciferase activities (RLA) in the lysed cells. For the wild type Pr8 contrast, p300 over-expression was able to super-drive the CM induced promoter activity. With mutation of the two functional AP-1 sites, only the upstream AP-1 site at -5.3 kb (Pr8-1u) showed the loss of p300 effect on CM inducibility. Mutation of the downstream AP-1 site at -5.1 kb did not alter p300 mediated effects (Pr8-1d) (Fig 3A). The double AP-1 mutant (1u +1d) showed a similar effect as the upstream AP-1 mutant (1u). Mutation of the NF-κB or Stat-1 sites in the enhancer region also did not affect p300 mediated inducibility (S3 Fig).

### In vivo binding of p300 to specific AP-1 proteins at -5.3 kb in the hiNOS enhancer

In order to validate that p300 binds to AP-1 proteins at -5.3 kb in vivo and to determine which AP-1 proteins were involved, ChIP assay was carried by using CM-stimulated A549 cells. Two specific DNA primers were designed from the hiNOS promoter spanning the -5.3 kb or -5.1 kb AP-1 sites. As shown in Fig 3B, Jun-D and Fra-2 proteins interacted with both -5.3 kb upstream and -5.1 kb downstream AP-1 sites. However, p300 only bound in vivo to the upstream -5.3 AP-1 site, which suggests that p300 formed a protein—protein complex with AP-1 proteins Jun-D and Fra-2. These findings are consistent with p300 mediated hiNOS transactivation at the hiNOS enhancer mainly through the upstream AP-1 site at -5.3 kb by forming a DNA-Protein complex.

### Long range DNA looping in hiNOS transcription bridged by p300

**Fig 4:**
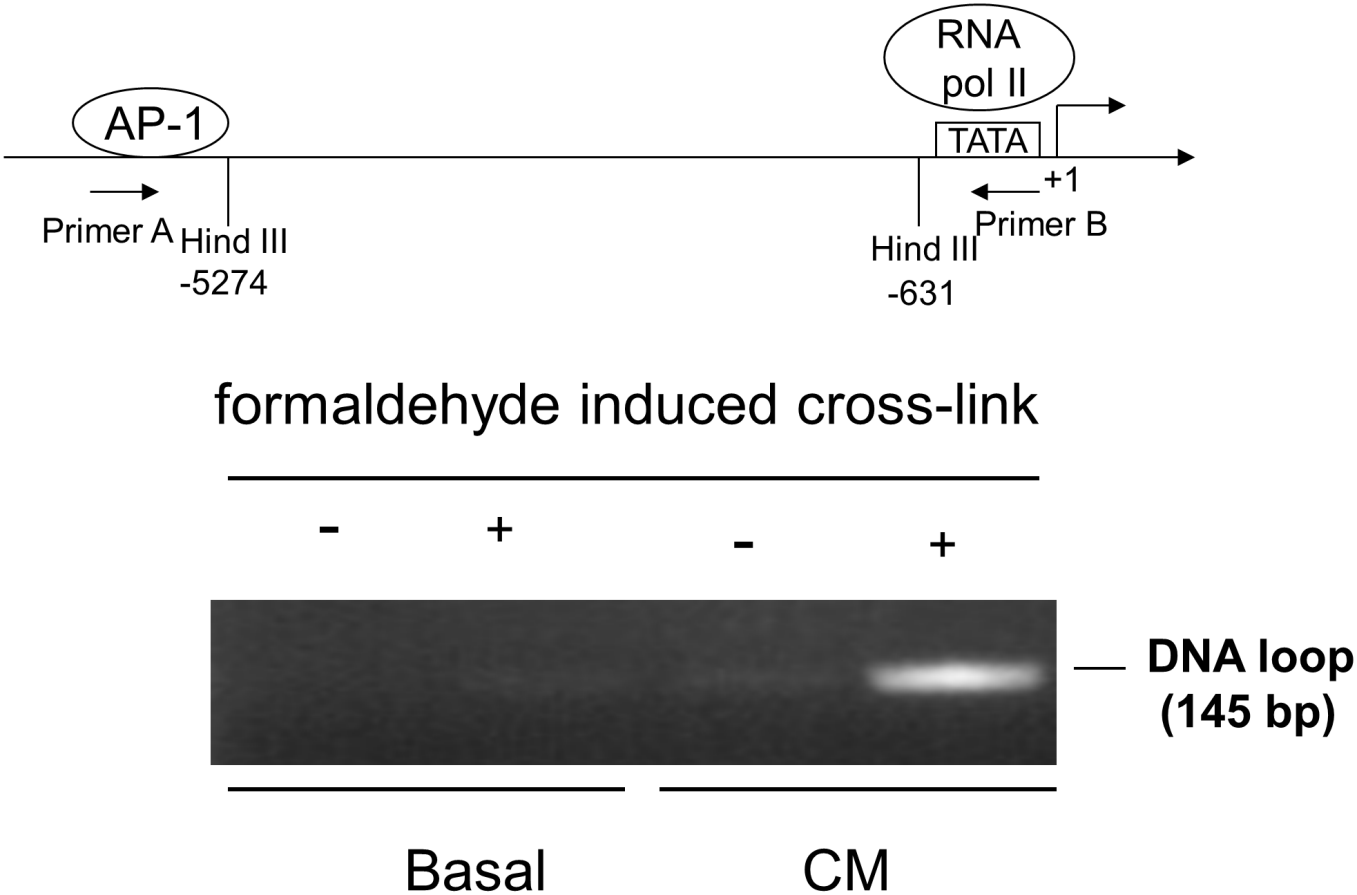
3C assay of DNA looping. Hind III restriction enzyme spliced at -5,274 and -631 bp in the hiNOS promoter. The gel of the 3C assay shown is representative of three experiments.

To show that the -5.3 kb AP-1 site in the hiNOS enhancer forms a DNA loop, we performed a 3C assay using restriction site Hind III at -5,274 bp and Hind III at -631 bp. The upstream -5.3 kb AP-1 site remains intact in the DNA, while the downstream AP-1 site at -5.1 kb is excised within the Hind III cutting zone. A DNA looping product was observed after CM stimulation in the DNA gel electrophoresis by 3C assay after formaldehyde induced cross-link (Fig 4). However, no DNA looping was observed in the basal groups.

**Fig 5:**
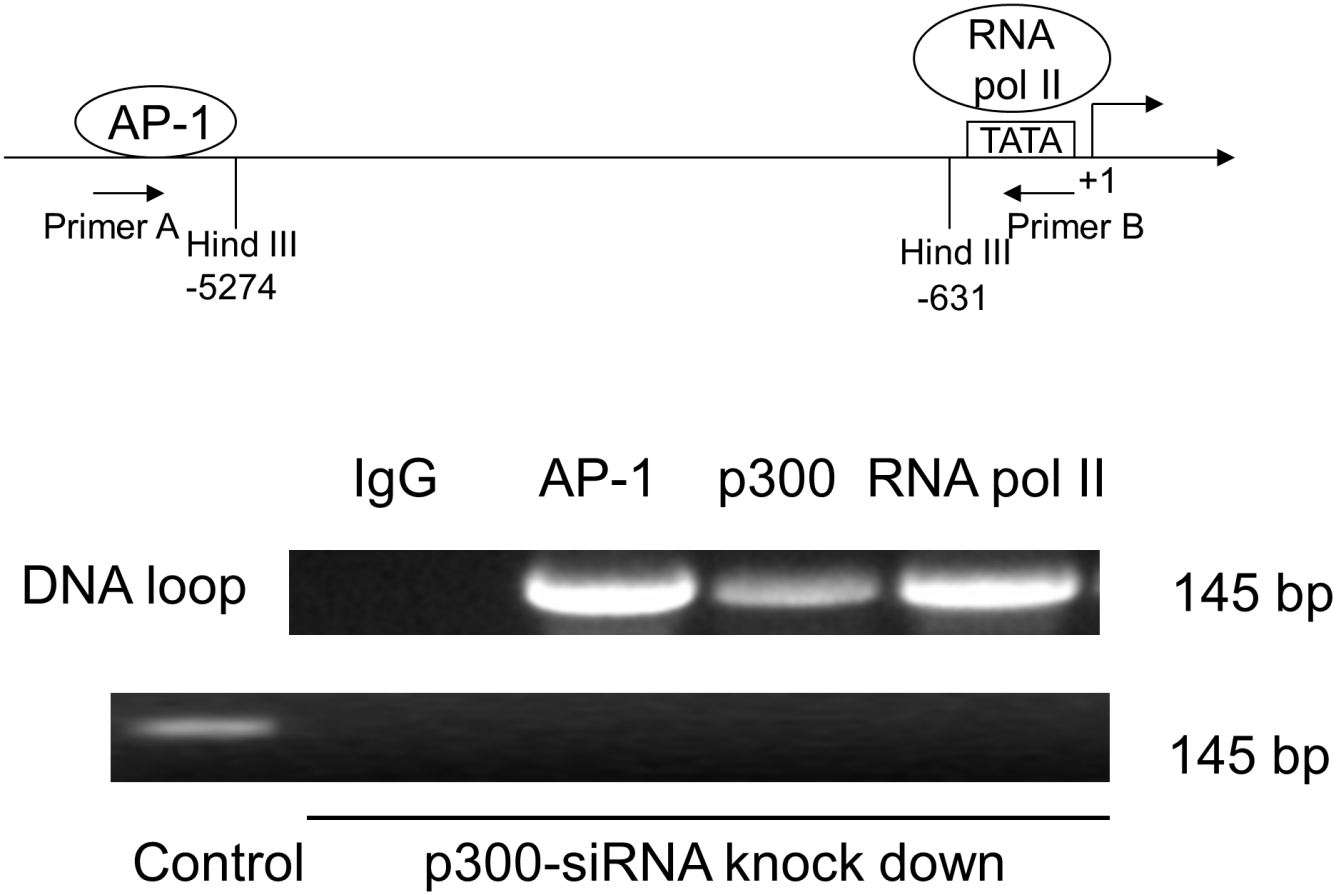
ChIP-Loop assay. Gel assay for AP-1, RNA pol II, and p300 binding. Schematic representation of AP-1 sites in the human iNOS promoter with relevant target sequences for Hind III restriction endonucleases and location of PCR primers. Ig G serves as negative control. Upper lane: without p300 siRNA treatment; Lower lane: with p300 siRNA treatment, p300 antibody with scrambled control siRNA serves as positive control. Gel assay shown is representative of three similar experiments.

To confirm that p300 protein was directly involved in the DNA loop complex, we performed ChIP loop assay by co-immunoprecipitating AP-1, p300, and RNA pol II which binds to the TATA box. Gel electrophoresis showed that DNA looping formed co-precipitated complexes with antibodies for AP-1, p300, and RNA Pol II, but not IgG which served as negative control (Fig 5). Furthermore, we performed an additional ChIP loop assay with p300-siRNA knockdown, and we were no longer able to detect the DNA loop band with co-immunoprecipitating AP-1, p300, or RNA pol II (Fig 5). This result indicates that the long range DNA looping in CM-treated A549 cells occurs via interactions between the AP-1 and TATA box sites. Moreover, AP-1, RNA Pol II, and p300 proteins are required for forming this long DNA loop.

## Discussion

The major and novel findings of our study are: 1) p300 can trans-activate human iNOS gene; 2) Upstream hiNOS enhancer at -5 kb and -6 kb is necessary for p300 CM induced transactivation; 3) p300 can mediate transactivation in heterologous minimal promoter; 4) CM induced a long range DNA looping between the AP-1 and TATA box sites in human iNOS promoter; 5) AP-1, RNA Pol II, and p300 proteins are required for forming this long DNA loop.

The initiation of transcription by RNA polymerase II usually requires the basal transcription machinery and sequence-specific promoter/enhancer-binding transcription factors. As a transcription coactivator, p300 can interact with a variety of transcription factors as well as with components of the basal transcriptional machinery, including TBP, TFIIB, TFIIE and TFIIF proteins. It may form a physical bridge or scaffold by connecting to the upstream enhancer sequence and downstream basal transcriptional machinery of a specific gene [20]. By interacting simultaneously with important upstream transcription factors and the core promoter region, p300 can stabilizes the transcription complex and activate gene transcription [21], [22].

p300 has been shown to play a major role in murine iNOS transcriptional activation. Deng and Wu have demonstrated that coactivator p300 regulates LPS-induced iNOS expression by increasing NF-κB binding and transactivation downstream in the murine iNOS promoter. Cao *et al*. have subsequently corroborated the role of p300 in iNOS expression using E1A, a specific adenovirus-derived inhibitor of p300 [23]. Another group has found that AP-1 c-Jun, NF-κB p65, and p300 protein complex requires p300 acetyltransferase activity for short range DNA looping in the setting of LPS stimulation [24]. However, all of the above studies are restricted to downstream murine iNOS promoter. Our current data is the original study to identify a specific role for p300 in the human iNOS promoter. We identify p300 protein-protein binding to far upstream -5.3 kb AP-1 site in the hiNOS enhancer region. Moreover, the AP-1 members in the hiNOS promoter that bind p300 are Jun D and Fra2 which are different than what has been shown in the murine AP-1 binding of c-Jun [16], [25].

Although our data (Fig 1A) showed that cytokines did not significantly induce p300 nuclear proteins in A549 cells or human hepatocytes, cytokines were shown to induce the changes in chromatin structure at human iNOS promoter in lung and liver cells [26]. Thus, we hypothesized that cytokines may remodel local chromatin structure and promote p300 to form a transcriptional complex for the initiation of human iNOS transcription. Our ChIP assay was able to detect in vivo binding of p300 to AP 1 protein in the hiNOS enhancer (Fig 3B). Our data support that p300 mediated hiNOS transactivation at the hiNOS enhancer mainly through the upstream AP-1 site at -5.3 kb by forming a DNA-Protein complex. It was further confirmed by p300 siRNA knock-down experiment with blocking its DNA loop formation. Therefore, what’s really important is the ability of p300 binding to AP1 proteins to form a transcriptional protein complex at hiNOS promoter, not the relatively endogenous expression level of p300 protein.

**Fig 6:**
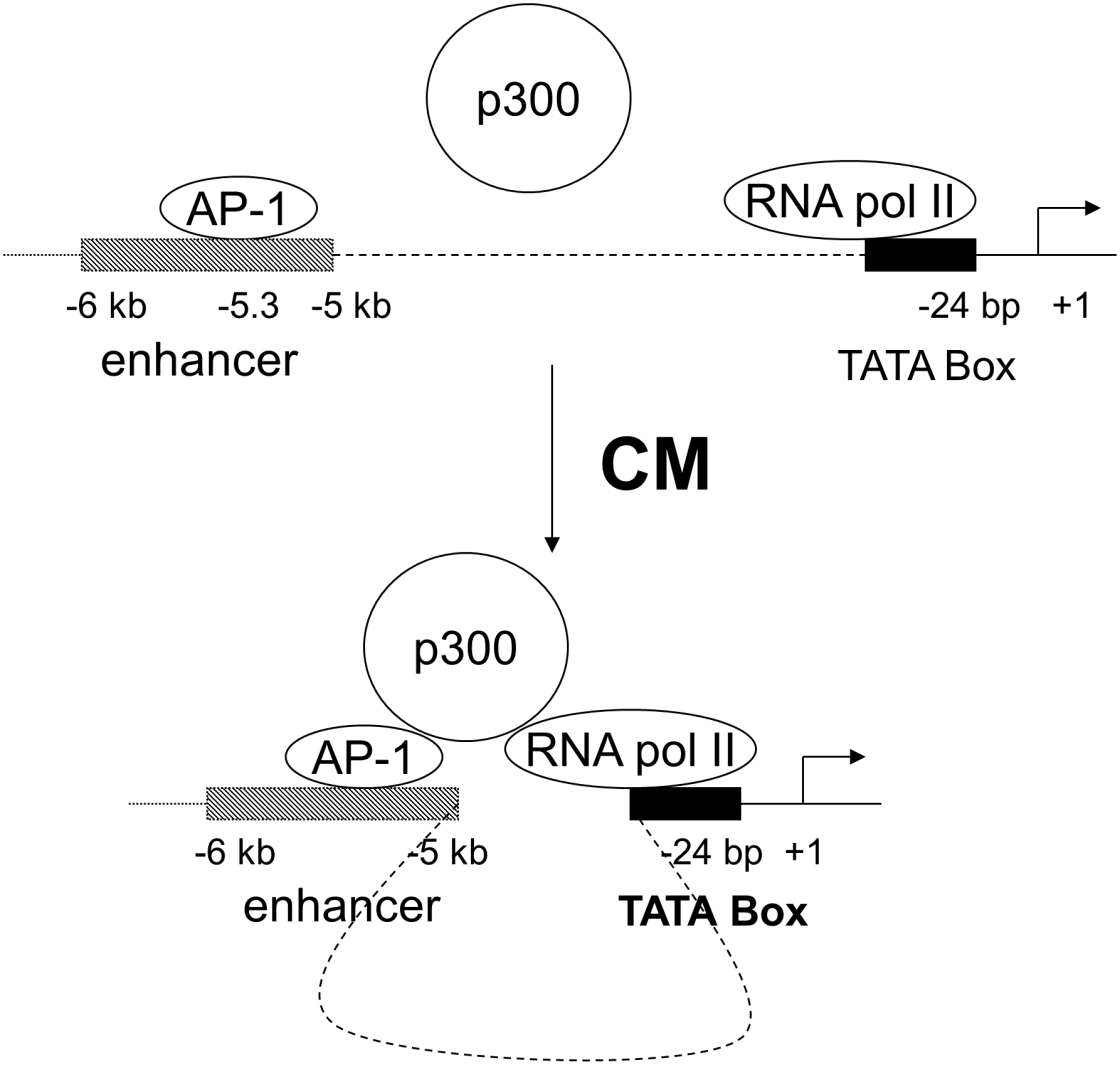
Schematic representation of potential interactions between the AP-1 site and RNA pol II in the human iNOS promoter with p300 in DNA loop formation.

Our results support that p300 has an important role in human iNOS gene transcription. Under cytokine stimulation, p300 interacts with Jun D, and Fra 2 proteins, thereby increasing AP-1 binding to its -5.3 kb response element in the human iNOS promoter. It also forms a complex with RNA Pol II at the TATA box of human iNOS core promoter. We demonstrate that long range DNA looping occurs at the human iNOS promoter. This looping is induced by cytokines and requires the presence of AP-1, Jun D, Fra2, and p300-associated basal transcriptional machinery. The distal AP-1 binding site at -5.3 kb interacts via p300 with the proximal TATA site to create this DNA loop to participate in CM induced hiNOS transcription (Fig 6). Taken together, these findings further underscore the complexity of the human iNOS gene transcriptional regulation.

## Supporting Information

S1 Fig Effect of p300 siRNA on endogenous p300 proteins in A549 cells. Vector p300 was overexpressed in the rescue group. Three similar Western blot experiments were quantified for p300 proteins after normalization with control group.
(TIF)

S2 Fig Effect of p300 knockdown with p300-siRNA on hiNOS mRNA expression. (TIF)

S3 Fig Mutagenesis analysis of NF-κB and Stat-1 sites at -5.5 kb or -5.8 kb of hiNOS enhancer region. (TIF)
