# Supplementary figures and images for: Up-Regulation of Human Inducible Nitric Oxide Synthase by p300 Transcriptional Complex
Source: PLoS One. 2016 Jan 11;11(1):e0146640. doi: 10.1371/journal.pone.0146640 (PMC4713468; doi:10.1371/journal.pone.0146640)

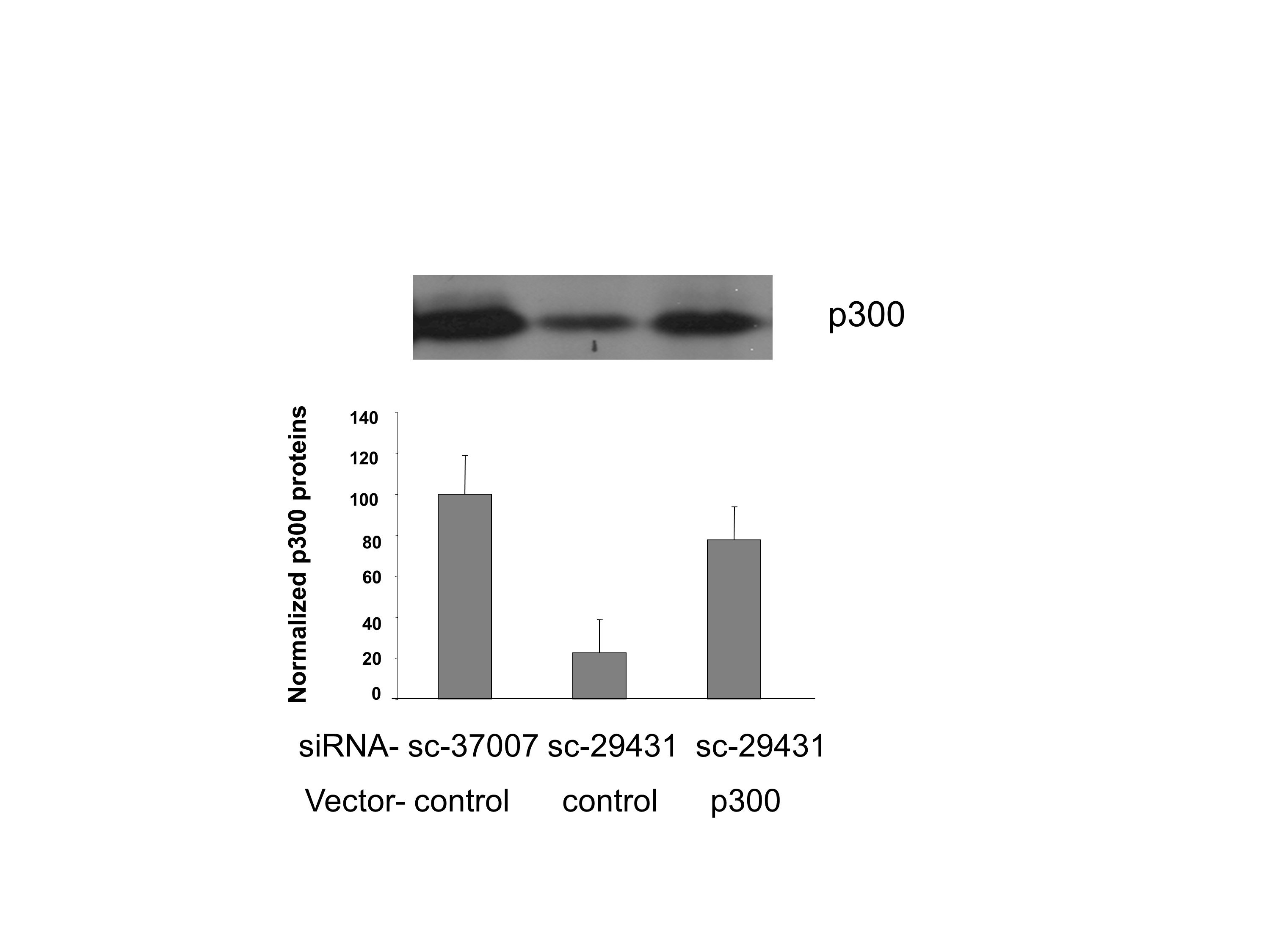

Supplement: S1 Fig — Vector p300 was overexpressed in the rescue group. Three similar Western blot experiments were quantified for p300 proteins after normalization with control group. (TIF) [file pone.0146640.s001.TIF]

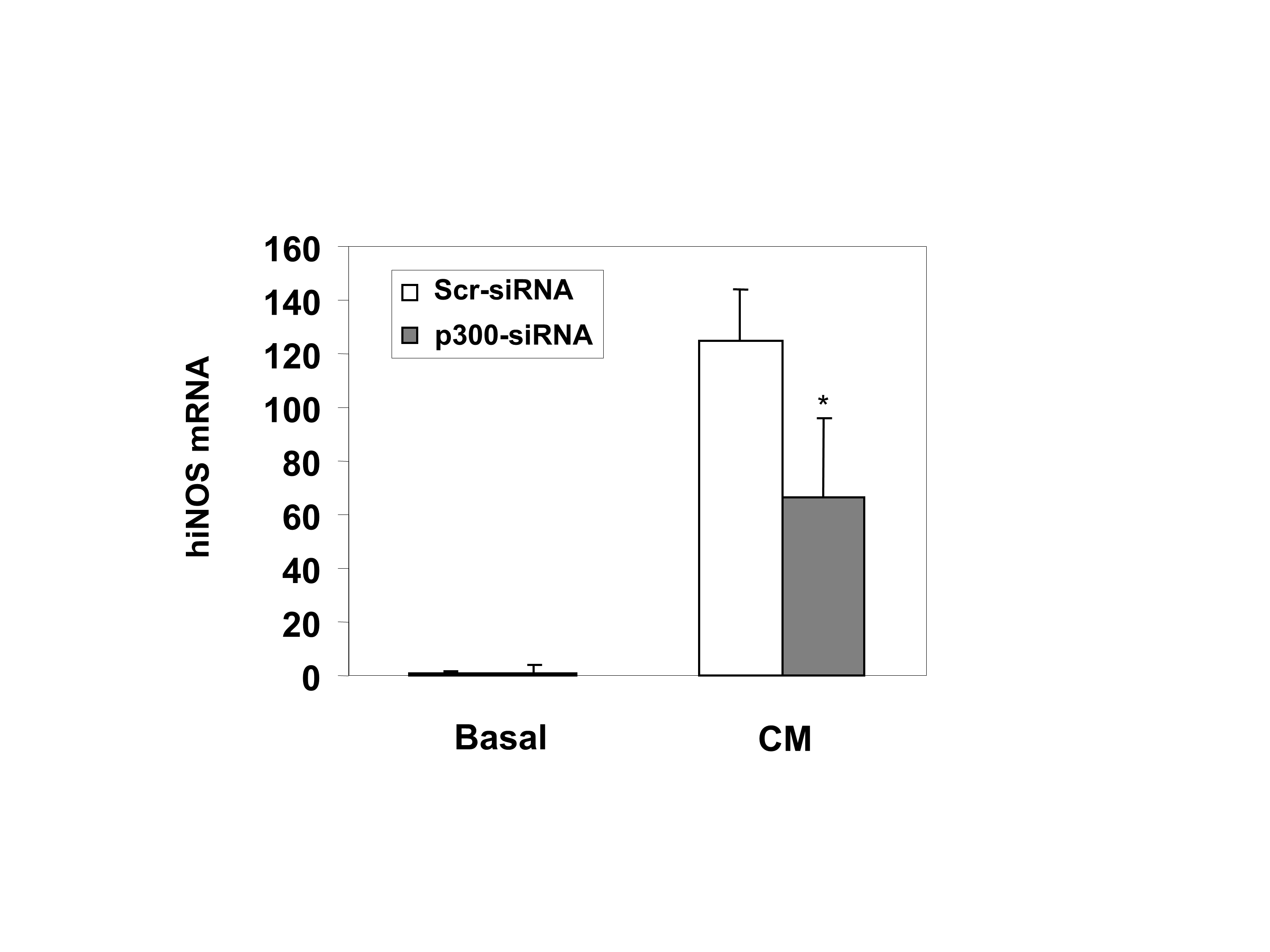

Supplement: S2 Fig — (TIF) [file pone.0146640.s002.TIF]

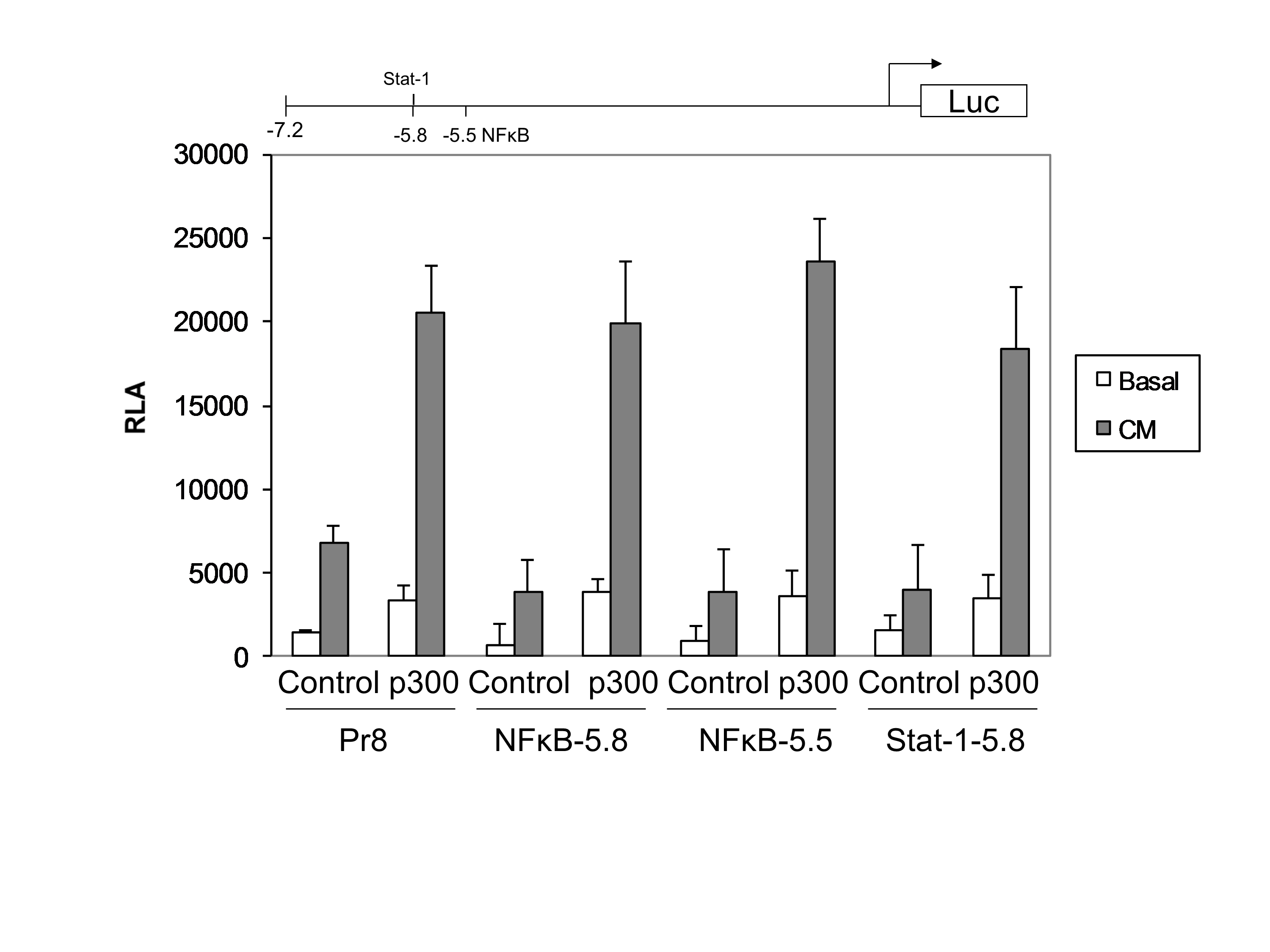

Supplement: S3 Fig — (TIF) [file pone.0146640.s003.TIF]
